# Unmet Health Care Use Among Socially Withdrawn Youth (Hikikomori) in South Korea: Cross-Sectional Survey Study

**DOI:** 10.2196/79043

**Published:** 2025-10-02

**Authors:** Eunjeong Choi, Seoyeong Choi, Sukyong Jang

**Affiliations:** 1 Department of Public Health, Graduate School, Yonsei University, Seoul, Republic of Korea; 2 Department of Biohealth Industry, Policy Analysis Division, Graduate School of Transdisciplinary Health Science, Yonsei University, 50 Yonsei-ro, Seodaemun-gu, Seoul, 03722, Republic of Korea; 3 Department of Health Policy and Management, Graduate School of Public Health, Yonsei University, Seoul, Republic of Korea

**Keywords:** mental health, social isolation, health services accessibility, health care disparities, health services needs and demand

## Abstract

**Background:**

Hikikomori, a condition of severe social withdrawal, is a global public health issue characterized by prolonged isolation. Despite its growing prevalence, little is known about the health care needs and use patterns of socially withdrawn youth.

**Objective:**

The study aimed to examine the association between hikikomori status and unmet health care use to inform targeted interventions.

**Methods:**

Data were obtained from the 2022 Korean Youth Living Conditions Survey, a nationally representative cross-sectional survey of 14,966 participants aged 19 to 34 years. Survey weights were applied to account for the sampling design. Hikikomori status was classified based on self-reported withdrawal behaviors, stratified by severity and duration. Unmet health care use in physical and mental health was assessed. Adjusted prevalence ratios (aPRs) with 95% CIs were estimated using generalized estimating equation models, and logistic regression was applied for subgroup analyses.

**Results:**

The weighted prevalence of perceived need for mental health services was 63.9% in the hikikomori group versus 50.5% in the non-hikikomori group (aPR 1.27, 95% CI 1.15-1.41). Unmet health care use was higher among individuals with hikikomori for physical care (aPR 3.33, 95% CI 2.16-5.13) and mental health care (aPR 4.46, 95% CI 2.92-6.81). Associations strengthened with greater severity and longer duration: for unmet mental health care use, aPRs were 4.14 (95% CI 2.64-6.49) for stage 1 and 9.52 (95% CI 3.67-24.65) for stage 2; by duration, aPRs were 2.57 (95% CI 1.11-5.96) for pre-hikikomori and 5.44 (95% CI 3.44-8.58) for hikikomori. Effect modification was observed by labor force participation, with higher risks among those not in the labor force (*P* for interaction <.05).

**Conclusions:**

Hikikomori is strongly associated with unmet health care use, particularly in mental health, with risks amplified by severity and duration. Tailored policies, including community-based outreach and remote health care interventions, are urgently needed to address these gaps.

## Introduction

### Background

The term “social isolation” is also used as a translation for the Japanese term “hikikomori,” which refers to individuals who have withdrawn from social interactions [1]. Hikikomori, initially defined in academic research, pertains to Japanese youth who withdraw from social interaction and primarily remain in their rooms, limiting their activities outside the home [1,2]. Although hikikomori was originally considered a unique cultural phenomenon in Japan during the 1990s [3], it has since emerged as a significant global issue [1,4-7]. Hikikomori is defined as remaining physically isolated in one’s home for at least 6 months, with individuals experiencing social isolation for at least 3 months proposed to be classified as “pre-hikikomori” [8,9]. Research conducted in Korea [10] and Hong Kong [11] used a shorter duration criterion (3 months) to define severe social withdrawal; therefore, defining the duration criteria for social withdrawal remains challenging.

In Japan, the estimated prevalence of hikikomori among individuals aged 15 to 39 years is approximately 2.3% [12]. In South Korea, recent survey data indicated a point prevalence of 2.4% in the national youth population, equivalent to an estimated 244,000 individuals [13]. Recent studies suggest that it may represent a broader global phenomenon. For example, analyses of the European Social Survey across 29 countries have estimated the prevalence of severe social withdrawal at approximately 1.7% to 1.8% [14]. Hikikomori is a global public health issue often accompanied by significant functional impairment and distress [9]. A considerable proportion of individuals diagnosed with hikikomori also exhibit concurrent mental health conditions, such as depressive disorders or schizophrenia [15]. Studies have consistently identified a high risk of suicide in individuals with hikikomori [8,16]. Social isolation and loneliness are also prevalent phenomena identified as independent risk factors for adverse cardiovascular and brain health [17] and are linked to various health behaviors, such as smoking cessation [18].

The importance of considering hikikomori in health care lies in their limited social interactions and restricted mobility, which may hinder their access to and use of health care resources. These characteristics make it difficult for individuals with hikikomori to meet their health care needs. Self-reported unmet health care needs have been widely adopted as an indicator of health care accessibility [19-21]. Self-reported unmet health care needs are strongly associated with socioeconomic status [22]. Moreover, many factors, such as income, ethnicity, and sexual minorities, hinder young people from accessing necessary mental health care [22-25]. However, empirical evidence regarding unmet health care use among individuals with hikikomori is lacking. Research includes studies examining treatment recommendations for hikikomori from the perspective of psychiatrists [4] and others exploring treatment preferences among individuals with hikikomori [7]. Nonetheless, there is a lack of research on perceived unmet health care use among individuals with hikikomori. To develop and apply adequate interventions for hikikomori, it is necessary to investigate the unmet health care use of individuals with hikikomori.

### Objectives

This study examined associations between hikikomori status and unmet health care use, as well as perceived need for mental health services, and assessed whether these associations varied by hikikomori severity and duration. The study also aimed to provide evidence to inform policies and interventions to improve the health of this population.

## Methods

### Data

This study used data from the 2022 Korean Youth Living Conditions Survey [13]. The Office of Government Policy Coordination in South Korea conducted the 2022 Youth Living Conditions Survey to collect baseline data for developing policies that aim to improve the quality of life for a diverse range of young people. The survey was conducted under the legal mandate of national youth policy legislation and is recognized as an official government statistical survey. This survey was conducted to understand the current state of young people’s lives and their characteristics, needs, and perceptions. Conducted biennially by the Korea Institute for Health and Social Affairs (KIHASA), this cross-sectional survey provides a comprehensive assessment across 8 domains: general characteristics, housing, health, education and training, employment, relationships and participation, social awareness, future planning, and economics. Data were collected through face-to-face interviews conducted by trained interviewers using structured questionnaires. To ensure a nationally representative sample of the South Korean youth population, a 2-phase probability sampling design was adopted, involving cluster sampling in the first stage and stratified cluster sampling in the second. The target population consisted of young adults aged 19 to 34 years residing in ordinary households across 17 administrative regions in South Korea. A stratified multistage sampling design was employed to ensure representativeness. Data were collected between July 18, 2022, and August 26, 2022.

### Participants

In total, 14,966 participants were included in the 2022 Korean Youth Living Conditions Survey. The study population was drawn from the 2020 Population and Housing Census Youth Statistics Register and comprised individuals aged 19 to 34 years as of January 1, 2022, who were members of ordinary households and were selected using a 2-stage sampling method. Ordinary households included private family households, while group quarters such as dormitories or institutions were excluded. The analytic sample thus consisted of all respondents who completed the survey.

### Hikikomori Status

Hikikomori status, the primary variable, was determined using a national youth life survey [13]. The Korean government classified individuals as hikikomori if they preferred staying at home based on their responses to questions about outdoor activities. The inclusion criteria included (1) staying home except for hobbies, (2) going out only to nearby stores, (3) staying entirely at home, or (4) rarely leaving their room.

### Hikikomori Severity and Duration

Hikikomori individuals were further categorized by severity and duration. Severity has 2 stages [13] where stage 1 (moderate isolation) involves leaving home for hobbies or nearby stores, while stage 2 (severe isolation) involves staying entirely at home or rarely leaving one’s room. Duration was classified as “pre-hikikomori” (<6 months of withdrawal) [9] or “hikikomori” (≥6 months of withdrawal) [26].

### Perceived Need for Mental Health Services

The perceived need for mental health services was a commonly assessed variable in mental health research [27,28]. Participants who reported recognizing the need for professional mental health counseling or treatment within the past year were coded as “yes,” whereas those who did not were coded as “no.”

### Assessment of Unmet Health Care Use

Measuring unmet health care use lacks a universal standard and is influenced by cross-cultural differences and survey methods. International surveys report varying degrees of unmet needs across countries [29]. Participants were asked if they had needed but not received medical care for physical health (excluding mental health) in the past year. For mental health care, they were asked about unmet needs for professional counseling within the same period [13].

### Other Covariates

To account for potential confounding variables associated with the independent and dependent variables, the analysis included the following covariates selected based on previous studies: demographic variables (age and sex); socioeconomic variables (household income quartile and labor force participation); and health-related variables (alcohol consumption, smoking, and physical activity) [19,30,31].

All selected covariates were categorized according to the study objectives:

1. Age groups: 19‐24, 25‐29, and 30‐34 years
2. Living status: living alone, living with parents, and living with others
3. Household income: high, low, and N/A. The participants were divided into quartiles on the basis of equivalized household income. For this analysis, the highest quartile was defined as “high” income and the lowest quartile was defined as “low” income; missing or nonresponse cases were coded as “N/A.”
4. Smoking status: the participants were self-reportedly classified as never, former, or current smokers.
5. Problem drinking: defined as the consumption of 6 or more units of alcohol per week for men and 4 or more units per week for women
6. Physical activity: coded as “yes” if participants reported engaging in regular exercise at least 3 times per week for health maintenance and “no” otherwise
7. Employment: the participants were categorized as employed (“yes”) if they reported working for pay for at least 1 hour during the previous week. Those who did not meet this criterion were classified as “no.”

### Statistical Analysis

The data were constructed using a multistage clustering and stratified sampling design to represent the South Korean population, based on data from the Youth Living Conditions Survey. To ensure national representativeness, all analyses incorporated survey sampling weights provided by the KIHASA, which account for the complex survey design, including unequal probabilities of selection and nonresponse adjustments. To estimate adjusted prevalence ratios (aPRs) and 95% CIs for the association between hikikomori status and unmet health care use, we used a generalized estimating equation model with a Poisson distribution and log link function. The generalized estimating equation approach was chosen because it provides robust variance estimates under a complex survey design and allows direct estimation of prevalence ratios, which are more interpretable than odds ratios when the outcome is common. In contrast, linear mixed models are primarily suited for longitudinal data with repeated measures, whereas our study is based on a cross-sectional survey [32]. The PR was adjusted for age, sex, region, family structure, household income, labor status, problematic drinking, smoking, and physical activity. The association between hikikomori status and the prevalence of unmet health care use, stratified by each level of covariates, was evaluated using logistic regression analysis. The PROC SURVEY procedure in SAS (version 9.4 M6; SAS Institute Inc) was used for all statistical analyses.

### Ethical Considerations

The study was reviewed by the institutional review board of Severance Hospital and determined to be exempt from full review (4-2024-1320). This study used deidentified secondary microdata from the KIHASA under a data use agreement. For the original data collection, informed consent was obtained by the data custodian; the secondary analysis was granted a waiver of additional consent because only deidentified data were analyzed. All datasets were anonymized with no direct identifiers, and only aggregate results are reported to protect privacy and confidentiality. No additional compensation was provided by this study (any compensation followed the original protocol). No identifiable images were included.

## Results

### Participant Characteristics

Table 1 shows the sociodemographic characteristics of the study population. Among 14,966 participants, the weighted prevalence of hikikomori was 2.9% (397/14,966). Compared with non-hikikomori individuals, those with hikikomori had substantially lower labor force participation (54/397, 15.5% vs 9434/14,569, 69%) and a higher proportion of low household income (187/397, 53.0% vs 5,022/14,569, 42.0%). They were also less physically active (112/397, 27.3% vs. 4783/14,569, 32.8%).

**Table 1.** Sociodemographic characteristics of participants. Values indicate unweighted counts (n) and weighted percentages (%) derived from survey weights (N=14,966).

Variables	Hikikomori status^a^						Standardized difference
	Total		Yes (397/14,966, 2.9%)		No (14,569/14,966, 97.1%)		
Age (y)^b^, n (%)							0.09
19-24	7195 (35.3)		179 (33.6)		7016 (35.3)		
25-29	4549 (34.1)		133 (36.7)		4416 (34.1)		
30-34	3222 (30.6)		85 (29.7)		3137 (30.6)		
Sex, n (%)							–0.06
Male	7171 (52.5)		180 (48)		6991 (52.6)		
Female	7795 (47.5)		217 (52)		7578 (47.4)		
Region, n (%)							–0.06
Metropolitan	4938 (53)		146 (59.6)		4792 (52.8)		
Nonmetropolitan	10,028 (47)		251 (40.4)		9777 (47.2)		
Living arrangement^c^, n (%)							0.26
Living alone	5355 (22.6)		99 (13.8)		5256 (22.9)		
Living with parents	7832 (57.5)		225 (58.8)		7607 (57.4)		
Living with others	1779 (19.9)		73 (27.4)		1706 (19.7)		
Labor^d^, n (%)							–1.23
No	5478 (32.5)		343 (84.5)		5135 (31)		
Yes	9488 (67.5)		54 (15.5)		9434 (69)		
Household income, n (%)							0.29
High	4319 (34.2)		107 (32)		4212 (34.3)		
Low	5209 (42.4)		187 (53)		5022 (42)		
N/A^e^	5438 (23.4)		103 (15)		5335 (23.7)		
Problem drinking^f^, n (%)							–0.18
No	12,802 (85.1)		362 (91.5)		12,440 (84.9)		
Yes	2164 (14.9)		35 (8.5)		2129 (15.1)		
Smoking status, n (%)							0.14
Current	2874 (20.1)		54 (14.3)		2820 (20.3)		
Former	1307 (10.1)		38 (9.5)		1269 (10.1)		
Never	10,785 (69.7)		305 (76.2)		10,480 (69.5)		
Physical activity^g^, n (%)							–0.10
No	10,071 (67.4)		285 (72.7)		9786 (67.2)		
Yes	4895 (32.6)		112 (27.3)		4783 (32.8)		

a Hikikomori status was defined as a result of the Korea Life Survey conducted by the Korea Institute for Health and Social Affairs in 2022.

b This study focused on individuals aged 19 to 34 years using data from the Youth Life Survey.

c Family structure was determined by the people living with each participant.

d Labor was determined by whether or not an individual had worked for pay for at least one hour in the past week.

e N/A: not available.

f Problem drinking is defined as 6 units of alcohol 2 or more times per week in men and 4 units of alcohol 2 or more times per week in women.

g Physical activity is defined as a regular physical activity for a week.

### Perceived Need for Mental Health Services

As shown in Table 2, hikikomori youth reported a greater perceived need for mental health services compared with their peers (259/397, 63.9% vs 7440/14,569, 50.5%), corresponding to a 27% higher likelihood of perceived need (aPR 1.27, 95% CI 1.15‐1.41). This association was stronger among individuals with more severe or prolonged withdrawal, with stage 2 participants showing the highest prevalence (91.1%) and risk (aPR 1.89, 95% CI 1.66‐2.15).

**Table 2.** Association between prevalence and perceived need for mental health services according to the hikikomori status, severity, and duration. All estimates were calculated using sampling weights and are presented as weighted prevalence (%) and weighted adjusted prevalence ratio (aPR). The unit of analysis was the individual participant.

Hikikomori status^a^	Participants, n	Prevalent participants, n	Weighted prevalence (%)	Weighted aPR^b^	Weighted 95% CI			*P* value
Model 1^c^								
No	14,569	7440	50.50	1	—^d^			—
Yes	397	259	63.9	1.27	1.15-1.41			<.001
Model 2^e^								
No	14,569	7440	50.50	1	—			—
Stage 1	375	240	62.3	1.24	1.12-1.38			<.001
Stage 2	22	19	91	1.89	1.66-2.15			<.001
Model 3^f^								
No	14,569	7440	50.50	1	—			—
Pre-hikikomori	140	77	50.5	1.01	0.82-1.26			.90
Hiki	257	182	71.0	1.41	1.27-1.57			<.001

a Hikikomori status was defined as a result of the Korea Life Survey conducted by the Korea Institute for Health and Social Affairs in 2022.

b Adjusted for age, sex, region, living arrangement, household income, labor, problem drinking, smoking, and physical activity. For the prevalence ratio, Poisson regression analysis was conducted.

c In model 1, hikikomori was determined by applying the criteria of the Youth Life Survey in 2022. Individuals were classified as hikikomori if they met any of the following criteria: (1) primarily staying at home and only going out for hobbies; (2) mostly staying at home and only going out to nearby convenience stores; (3) staying in their own room but not going outside the house; or (4) rarely leaving their own room.

d Not applicable.

e In model 2, those identified as hikikomori were further classified into 2 stages according to the severity of hikikomori status. Stage 1 was characterized by individuals who primarily confined themselves to their homes, venturing out only for hobbies or to nearby convenience stores. Individuals in stage 2 demonstrated more severe isolation, as they either remained in their homes or rarely left their rooms.

f In model 3, those identified as hikikomori were further classified into 2 groups based on duration of hikikomori status: *pre-hikikomori* for those who had a history of social withdrawal of less than 6 months [9] and *hikikomori* for those who had experienced social withdrawal for 6 months or longer [26].

### Unmet Health Care Use

Table 3 demonstrates that hikikomori status was strongly associated with unmet health care use. For physical health, the weighted prevalence was 8.6% among hikikomori youth compared with 2.9% among nonhikikomori (aPR 3.33, 95% CI 2.16‐5.13). For mental health, the corresponding figures were 10.3% versus 2.6% (aPR 4.46, 95% CI 2.92‐6.81). The association was particularly pronounced in stage 2 hikikomori, where the prevalence of unmet mental health care use reached 22.1%. Finally, Table 4 shows that the association between hikikomori and unmet physical health care use varied by labor force participation (for *P* interaction=.008).

**Table 3.** Association between hikikomori status and prevalence of unmet health care use. All estimates were calculated using sampling weights and are presented as weighted prevalence (%) and weighted adjusted prevalence ratio (aPR). The unit of analysis was the individual participant.

Hikikomori status^a^	Participants, n	Unmet health care use												
		Physical health						Mental health						
		Prevalence, n	Weighted prevalence (%)	Weighted aPR^b^	Weighted 95% CI		*P* value	Prevalence, n	Weighted prevalence (%)	Weighted aPR^b^	Weighted 95% CI			*P* value
Model 1^c^														
No	14,569	427	2.90	1	—^d^		—	383	2.60	1	—			—
Yes	397	32	8.6	3.33	2.16-5.13		<.001	40	10.3	4.46	2.92-6.81			<.001
Model 2^e^														
No	14,569	427	2.90	1	—^d^		—	383	2.60	1	—			—
Stage 1	375	30	8.8	3.37	2.17-5.26		<.001	36	9.6	4.14	2.64-6.49			<.001
Stage 2	22	2	6	2.48	0.49-12.55		.27	4	22	9.52	3.67-24.65			<.001
Model 3^f^														
No	14,569	427	2.90	1	—		—	383	2.60	1	—			—
Pre-hikikomori	140	3	1.8	0.78	0.25-2.47		.67	9	5.7	2.57	1.11-5.96			.03
Hikikomori	257	29	12.2	4.55	2.89-7.15		<.001	31	12.7	5.44	3.44-8.58			<.001

a Hikikomori status was defined as a result of the Korea Life Survey conducted by the Korea Institute for Health and Social Affairs in 2022.

b Adjusted for age, sex, region, living arrangement, household income, labor, problem drinking, smoking, and physical activity. For the prevalence ratio, Poisson regression analysis was conducted.

c In model 1, hikikomori was determined by applying the criteria of the Youth Life Survey in 2022. Individuals were classified as hikikomori if they met any of the following criteria: (1) primarily staying at home and only going out for hobbies; (2) mostly staying at home and only going out to nearby convenience stores; (3) staying in their own room but not going outside the house; or (4) rarely leaving their own room.

d Not applicable.

e In model 2, those identified as hikikomori were further classified into 2 stages according to the severity of hikikomori status. Stage 1 was characterized by individuals who primarily confined themselves to their homes, venturing out only for hobbies or to nearby convenience stores. Individuals in stage 2 demonstrated more severe isolation, as they either remained in their homes or rarely left their rooms.

f In model 3, those identified as hikikomori were further classified into 2 groups based on duration of hikikomori status: “pre-hikikomori” for those who had a history of social withdrawal of less than 6 months [9] and “hikikomori” for those who had experienced social withdrawal for 6 months or longer [26].

**Table 4.** Association of hikikomori status^a^ prevalence with unmet health care use stratified by each level of covariates.

Variables	Physical health							Mental health						
	Non-hikikomori weighted OR^b^ (reference)	Hikikomori weighted OR	95% CI			*P* value	*P* for interaction^c^	Non-hikikomori weighted OR (reference)	Hikikomori weighted OR	95% CI			*P* value	*P* for interaction
Age (y)^d^														
19‐24	1	3.33	1.64-6.77			.001	.18	1	4.65	1.88-11.53			<.001	.60
25-34	1	3.19	1.74-5.85			.001	—^e^	1	4.82	2.70-8.58			<.001	—
Sex														
Male	1	3.47	1.49-8.07			.004	.74	1	4.74	2.21-10.44			<.001	.71
Female	1	3.55	1.99-6.33			<.001	—	1	5.01	2.73-9.19			<.001	—
Region														
Metropolitan area	1	2.85	1.40-5.82			.004	.42	1	4.50	2.26-8.96			<.001	.56
Nonmetropolitan area	1	4.82	2.60-8.93			<.001	—	1	6.04	3.43-10.62			<.001	—
Family structure^f^														
Living alone	1	2.48	0.85-7.20			.10	.37	1	3.26	1.20-8.86			.02	.39
Living with others	1	3.84	2.24-6.58			<.001	—	1	5.52	3.19-9.53			<.001	—
Labor^g^														
No	1	3.97	2.27-6.94			<.001	.008	1	5.43	1.93-15.30			.001	.89
Yes	1	0.61	0.14-2.57			.49	—	1	4.60	2.70-7.81			<.001	—
Problem drinking^h^														
No	1	2.96	1.76-4.97			<.001	.08	1	5.14	3.09-8.56			<.001	.48
Yes	1	18.08	5.60-58.43			<.001	—	1	3.35	0.63-17.75			.16	—
Smoking status														
Yes	1	12.77	3.63-44.99			<.001	.24	1	7.89	2.55-24.40			<.001	.33
No	1	4.47	2.64-7.58			<.001	—	1	3.13	1.84-5.33			<.001	—
Physical activity^i^														
No	1	3.33	1.91-5.81			<.001	.89	1	4.78	1.96-11.69			.001	.87
Yes	1	4.56	1.77-11.79			.002	—	1	5.17	2.90-9.21			<.001	—

a Hikikomori status was defined as a result of the Korea Life Survey conducted by the Korea Institute for Health and Social Affairs in 2022.

b OR: odds ratio.

c The *P* for interaction tests whether the effect of the treatment differs significantly across subgroups. Unlike the subgroup-specific *P* values, which assess the effect within each subgroup, the *P* for interaction evaluates whether the differences in effects between subgroups are statistically significant.

d This study focused on individuals aged 19 to 34 years using data from the Youth Life Survey.

e Not applicable.

f Family structure was determined by the people living with each participant.

g Labor was determined by whether or not an individual had worked for pay for at least 1 hour in the past week.

h Problem drinking is defined as 6 units of alcohol 2 or more times per week in men and 4 units of alcohol 2 or more times per week in women.

i Physical activity is defined as a regular physical activity for a week.

## Discussion

### Principal Findings

This study found a significant association between hikikomori and a higher likelihood of the perceived need for mental health services compared to those without hikikomori. This association was more prominent in individuals with severe hikikomori and a longer duration of hikikomori. In addition, this study found a significant association between hikikomori and a higher prevalence of unmet health care use in both physical and mental health. This association became more pronounced as the severity and duration of hikikomori increased. Moreover, the prevalence of unmet health care use in mental health was higher than in physical health.

In this study, individuals with hikikomori were more likely to perceive a need for mental health care than those without hikikomori, meaning they recognized their needs independently. This tendency also increased with greater hikikomori severity and duration. The perceived need for mental health care is known to influence the use of services [33], and a previous study reported that individuals with mental health conditions who do not believe they require assistance are less likely to seek treatment [34]. However, despite recognizing their needs, hikikomori individuals reported substantially higher levels of unmet health care use, as seen in Table 3. Because of their unique behavioral patterns, individuals with hikikomori may face significant barriers to accessing health care services, even when they recognize their deteriorating health. Future strategies should include home-visiting health services or remote health care services to address the unmet health care needs of hikikomori and facilitate easier access to care.

Our study is significant because, in contrast to previous research, which primarily focused on comparing the mental health status of individuals with hikikomori [35-37], the results of this study highlight the challenges faced by individuals with hikikomori in accessing health care use compared with those of individuals without hikikomori. We investigated the association between hikikomori status and health care use by stratifying individuals based on hikikomori severity, determined by levels of isolation derived from staying at home. Our findings revealed a gradual increase in the risk of unmet health care use with an increase in hikikomori severity. Individuals with the most severe form of hikikomori experienced a substantial increase in the risk of unmet health care use in mental health, although the number of participants in this specific subgroup was limited. This finding can be interpreted as follows: compared to mild hikikomori, who sometimes go out for hobbies or to the market, severe hikikomori mostly stay in their homes (or rooms), preventing them from using health care services. Furthermore, unmet health care use in mental health was more prevalent among individuals with the most severe hikikomori than in physical health. Based on these results, the more severe the stage of hikikomori, the more urgent the need to address their unmet health care use.

The results of this study also revealed a gradual increase in the risk of unmet health care use in both physical and mental health with increasing duration of hikikomori status. This result is similar to that of a study examining treatment preferences in a sample of individuals with hikikomori who expressed a desire to seek treatment for their condition compared to pre-hikikomori individuals (people who withdrew for less than 6 months) [7]. Individuals with long-term social withdrawal tend to have restricted social activities and behavioral problems [38]. In this context, the unmet health care use of individuals with hikikomori for long-term periods must be urgently addressed to provide them with appropriate health services. The subgroup analysis showed that the impact of hikikomori on unmet physical health care use was especially pronounced among those outside the labor force, suggesting that economic inactivity is an important barrier to accessing physical health services. Prior studies have similarly reported greater unmet needs among unemployed or economically inactive individuals [39], consistent with our findings in socially withdrawn youth.

In this study, individuals with hikikomori, severe hikikomori, and longer durations of hikikomori were more likely to have unmet mental and physical health use. Individuals with hikikomori who choose not to seek help experience more negative consequences [15,35]. On the basis of these findings, individuals with hikikomori should be treated with adequate interventions to prevent the worsening of health conditions. Considering their behavioral patterns, future interventions for hikikomori may benefit from home-visit programs led by health professionals. This approach could be particularly effective in improving the management systems for individuals with hikikomori [7,10]. In addition, the findings of this study showed that the likelihood of unmet health care use in hikikomori was greater than in nonhikikomori, and this trend was stronger when hikikomori severity and duration worsened. This suggests that health care providers should develop and implement patient-centered programs based on individual hikikomori status. Given the significant trend observed in mental health compared to physical health, a deeper consideration of health assessments and interventions specifically tailored for individuals with hikikomori is warranted.

Although this study did not aim to identify causal mechanisms, previous research has suggested several potential contributors to hikikomori, including individual vulnerabilities, family dynamics, and broader social factors such as academic pressure, insecure employment, and housing instability [4,8,16]. In addition, stigma surrounding mental illness and social withdrawal may discourage individuals from seeking help, reinforcing isolation. Structural barriers, including shortages of mental health professionals and limited community-based services, further restrict access [40]. Addressing these challenges will require not only expanding conventional services but also integrating innovative approaches such as remote counseling and health care [23,41]. Strengthening the workforce, reducing regional disparities, and evaluating the effectiveness of digital strategies will be essential to better meet the needs of socially withdrawn youth.

### Strengths and Limitations

Our study has several limitations. First, the cross-sectional design used in this study meant that the data were collected at a single time point. This limits our ability to establish clear causal associations, which is why the results indicate associations rather than definitive causal relationships. Reverse causality is a common drawback of cross-sectional studies. However, it can be argued that the prevalence of hikikomori is more likely to be a causal factor influencing the unmet health care use status of individuals with hikikomori, rather than vice versa. Second, the survey used in this study did not consider participants’ motivation to seek health care. Therefore, we could only examine the prevalence of hikikomori and unmet health care use. However, we adjusted for socioeconomic characteristics, such as income, labor, region, and family structure, so that significant factors affecting health care use could be considered. Third, this study relied on self-reported survey responses, which may be subject to recall bias. While the young age of participants may mitigate this issue, the possibility of such bias cannot be entirely excluded and should be considered when interpreting the results. Fourth, unmet health care use was defined as situations in which participants reported needing but not receiving medical care. The survey did not capture whether individuals actively sought help, including alternative modes such as online consultations. Finally, the data were collected from one country. Cross-cultural and methodological differences can introduce bias into the assessment of unmet health care needs, complicating cross-country comparisons.

Despite its limitations, this study has several strengths. To our knowledge, it is the first quantitative analysis to link hikikomori status with unmet health care use. The data, representing the entire Korean youth population, enable extrapolation of findings to a broader context. Reliability was enhanced by collecting self-reported data through in-person interviews. The findings can inform policy discussions and support the integration of interventions for withdrawal behaviors and medical treatment.

### Conclusions

This study suggests that individuals with hikikomori face challenges in accessing and seeking mental health care services. It demonstrated a remarkable association between hikikomori status and unmet health care use, emphasizing the profound impact of hikikomori status on the prevalence of unmet health care use across varying levels of severity and duration. It may be beneficial for providers and policymakers to explore the potential of home-visit services or technology-assisted services, such as telehealth, as supplementary measures to better understand unmet health care use and provide more accessible care options.

## Abbreviations

aPR: adjusted prevalence ratio
KIHASA: Korea Institute for Health and Social Affairs
